# Bearing Fault Diagnosis Based on an Enhanced Image Representation Method of Vibration Signal and Conditional Super Token Transformer

**DOI:** 10.3390/e24081055

**Published:** 2022-07-31

**Authors:** Jiaying Li, Han Liu, Jiaxun Liang, Jiahao Dong, Bin Pang, Ziyang Hao, Xin Zhao

**Affiliations:** 1 National & Local Joint Engineering Research Center of Metrology Instrument and System, Hebei University, Baoding 071002, China; lijiaying2020hbu@163.com (J.L.); liuhanezhou@163.com (H.L.); jiaxun0118@163.com (J.L.); dongjiahao200006@163.com (J.D.); haoziyang@hbu.edu.cn (Z.H.); zhaoxinzj@hbu.edu.cn (X.Z.); 2 Hebei Technology Innovation Center for Lightweight of New Energy Vehicle Power System, Hebei University, Baoding 071002, China; 3 College of Quality and Technical Supervision, Hebei University, Baoding 071002, China

**Keywords:** multipoint envelope L-kurtosis, Vision Transformer, fault visualization, rolling bearing, fault diagnosis

## Abstract

Multipoint Optimal Minimum Entropy Deconvolution Adjusted (MOMEDA) is an advanced deconvolution method, which can effectively inhibit the interference of background noise and distinguish the fault period by calculating the multipoint kurtosis values. However, multipoint kurtosis (MKurt) could lead to misjudgment since it is sensitive to spurious noise spikes. Considering that L-kurtosis has good robustness with noise, this paper proposes a multipoint envelope L-kurtosis (MELkurt) method for establishing the temporal features. Then, an enhanced image representation method of vibration signals is proposed by employing the Gramian Angular Difference Field (GADF) method to convert the MELkurt series into images. Furthermore, to effectively learn and extract the features of GADF images, this paper develops a deep learning method named Conditional Super Token Transformer (CSTT) by incorporating the Super Token Transformer block, Super Token Mixer module, and Conditional Positional Encoding mechanism into Vision Transformer appropriately. Transfer learning is introduced to enhance the diagnostic accuracy and generalization capability of the designed CSTT. Consequently, a novel bearing fault diagnosis framework is established based on the presented enhanced image representation and CSTT. The proposed method is compared with Vision Transformer and some CNN-based models to verify the recognition effect by two experimental datasets. The results show that MELkurt significantly improves the fault feature enhancement ability with superior noise robustness to kurtosis, and the proposed CSTT achieves the highest diagnostic accuracy and stability.

## 1. Introduction

The rolling bearing is one of the most crucial parts of rotating machinery, which is widespread in industrial applications [1,2]. Due to the harsh working environment and variable heavy loads, many types of faults are likely to occur in rolling bearings, which may cause inestimable work accidents and financial losses. Therefore, accurate fault diagnosis of the rolling bearings is of great significance for ensuring mechanical system security and operational stability [3,4].

With the continuous development of artificial intelligence technology in the industrial field, diagnosis methods based on machine learning are universally used in the intelligent fault diagnosis of rotating machinery [5]. However, traditional machine learning methods need to manually set internal parameters, which have high requirements for background knowledge and professional experience. Meanwhile, the traditional machine learning methods are unable to adaptively learn the extracted signal features; thus, their recognition ability is limited. In order to address these issues, deep learning methods have been pioneered in fault diagnosis. Due to the powerful modeling and image feature extraction capabilities of deep learning methods, many previous studies have converted one-dimensional vibration signals into two-dimensional images as input for deep learning models. He et al. [6] processed the sensor data by the method of short-time Fourier transform (STFT) to obtain a spectrum image. Tao et al. [7] applied the short-time Fourier transform (STFT) to convert raw vibration signals into images. Shao et al. [8] generated a visual image of the raw signal using continuous wavelet transform (CWT). Wang et al. [9] obtained the 2D signal representation maps by synchro-extracting transform (SET). However, most of these signal-to-image methods heavily rely on expert experience to set appropriate internal parameters. According to the problem, some researchers have introduced the Gramian Angular Field (GAF) method to convert signals into images without selecting parameters [10]. Tang et al. [11] decomposed the vibration signals to gain the appropriate signal components and converted them into images by GAF. Han et al. [12] compared GAF with Markov Transition Field (MTF) and verified the superiority of GAF in information preservation. As a type of GAF, the Gramian Angular Difference Field (GADF) obtains a matrix by calculating the trigonometric difference between each point. It maintains the temporal dependency and preserves abundant features with polar coordinates. Therefore, GADF is employed to transform the vibration signals into images in this paper.

Due to the complex environment and the influence of vibration information from other mechanical components, the bearing fault vibration signal collected by the sensor contains background noise, which affects the accuracy of the fault diagnosis [13,14]. Bearing fault features can be extracted by performing optimal filtering on the signal to obtain obvious periodic impact components. Moreover, the collected bearing fault signal can be seen as the convolution of the impact signal with the transmission path, and the fault impact signal can be extracted by a deconvolution process [15]. Endo et al. [16] introduced the minimum entropy deconvolution (MED) to improve the ability to diagnose gear tooth faults, and it achieved great performance. The MED algorithm can only extract individual impulse features and may have spurious impulse components. Moreover, the iterative method of MED is complex, and the efficiency of finding the optimal filter is low. Considering the drawbacks of MED, McDonald et al. [17] proposed the maximum correlated kurtosis deconvolution (MCKD) by designing the correlated kurtosis norm as the target function of the filtering. Wang et al. [18] denoised the vibration signal by MCKD and effectively emphasized periodic impulses. Jia et al. [19] incorporated MCKD and an improved spectrum kurtosis to diagnose the early fault of bearings. Although MCKD can extract more impulse components, it can still only extract a limited number of impulses. In addition, the setting of MCKD internal parameters depends on prior knowledge, which means that noise reduction is only effective when the parameters are selected appropriately. In order to address the issues of the above two methods, Multipoint Optimal Minimum Entropy Deconvolution Adjusted (MOMEDA) method was developed [20]. Due to the unpredictability of the bearing fault period in practical engineering, MOMEDA deconvolves the signals of different preset target periods by presetting a period range, and the multipoint kurtosis (MKurt) is obtained by calculating the kurtosis of filtered signals. When the bearing component fails, the multipoint kurtosis spectrum will have significant peaks at the bearing fault period, as well as its harmonics, to reflect the fault information of the component. McDonald et al. [20] successfully applied MOMEDA to the fault detection of the gearbox. However, due to the kurtosis being sensitive to accidental pulses and less robust against noise, multipoint kurtosis might lead to the wrong indication when processing signals containing accidental pulses and heavy noise [21]. Considering that L-kurtosis is more robust to the spurious noise spikes compared with kurtosis, this paper develops a method for establishing temporal features of multipoint envelope L-kurtosis (MELkurt), and it is combined with GADF to propose an enhanced image representation method of vibration signals.

Due to the powerful performance of feature learning and extracting, intelligent diagnosis methods based on deep learning have been applied to various engineering areas [22]. In particular, models based on convolutional neural network (CNN) have been widely researched to solve the problems of bearing fault diagnosis [23,24]. Wang et al. [25] combined the squeeze-and-excitation (SE) network and CNN to propose SE-CNN, while using symmetrized dot pattern (SDP) images of vibration signals as input. Wen et al. [26] designed a new Transfer CNN (TCNN) and incorporated the architecture of TCNN with Visual Geometry Group 19 (VGG-19). Yao et al. [27] introduced the butterfly-transform (BFT) module to MobileNet V3 and proposed BFT-MobileNet V3, which achieved better diagnosis accuracy with less computation. Chen et al. [28] proposed a fault diagnosis method by incorporating Cyclic Spectral Coherence (CSCoh) with CNN, which effectively improved the recognition accuracy of bearing faults. CNN-based models have been successfully implemented for variable fault diagnosis issues and have achieved great success in previous studies. However, CNN-based models are weak at learning relationships between different pixel regions and rely on more convolutional layers when capturing global information. If the background noise is enhanced or the application scenario changes, the diagnostic accuracy and stability of CNNs will be reduced due to the lack of transfer capability. Motivated by the remarkable achievements of the transformer architecture models in natural language processing, many researchers have introduced transformer-based models to image processing. Currently, the transformer-based models have shown excellent transfer and modeling capabilities. To extend transformer-based models to the field of bearing fault diagnosis, this paper introduced the Vision Transformer (ViT) and enhanced its performance [29]. First, to overcome the shortcomings of ViT in modeling links between different local areas, we introduced the Super Token Transformer block and Super Token Mixer (STM) module [30]. Second, Conditional Positional Encoding (CPE) is incorporated into the designed model to improve the generalization ability [31]. Therefore, we proposed a novel deep learning method named Conditional Super Token Transformer (CSTT).

In this work, a novel intelligent diagnosis approach is established based on an enhanced vibration signal image representation method and CSTT. The MOMEDA is combined with the designed Multipoint Envelope L-Kurtosis to enhance the fault features of vibration signals. Then, GADF is applied to translate the enhanced signals into images in order to obtain distinguishing feature representations of different bearing faults. The proposed Conditional Super Token Transformer is utilized to recognize rolling bearing diagnosis fault types by taking advantage of its feature extraction capability.

The organization of this paper is as follows. The principles of MOMEDA and GADF are described in Section 2. In addition, the details of the designed Multipoint Envelope L-Kurtosis are introduced in this section. Section 3 introduces the proposed CSTT and its theoretical background. The proposed bearing fault diagnosis framework, based on the enhanced vibration signal image representation method and CSTT, is presented in Section 4. In Section 5, the proposed method is validated, and comparisons are carried out by using two different datasets. Finally, main conclusions are summarized in Section 6.

## 2. Enhanced Vibration Signal Image Representation Method

### 2.1. Multipoint Envelope L-Krtosis

Rolling bearings usually operate in a complex and harsh environment, which includes strong noise interference. Therefore, it is essential to extract the bearing fault impulses from raw vibration signals for timely and accurate bearing fault diagnosis. MOMEDA is a non-iterative deconvolution method for finding the optimal filter that minimizes the effect of noise on the vibration signal, thus achieving an accurate reconstruction of the original signal. The vibration signal ***x***(*n*) collected by the sensor can be expressed as:

(1) x(n)=h(n)∗y(n)+q(n)

where ***y***(*n*) represents the impact signal, ***h***(*n*) indicates the transfer function, and ***q***(*n*) is noise.

The deconvolution process is defined as:

(2) $$y=f*x=\sum_{k=1}^{N-L}f_{k}x_{k+L-1},\;k=1,\;2,\;\ldots ,\;N-L$$

where *k* represents the total sampling points, and *L* defines the filter length.

Based on the features of periodic pulse signals in vibration signals, MOMEDA obtains the optimal filter by computing the maximum value of the multipoint D-norm as follows:

(3) $$MDN(y,t)=\frac{1}{\left\|t\right\|}\frac{t^{T}y}{\left\|y\right\|}$$

(4) $$MOMEDA(y,t)=\underset{f}{\max}MDN\left(y,t\right)=\underset{f}{\max}\frac{t^{T}y}{\left\|y\right\|}$$

where ***t*** is the target vector that defines the positions and weights of the target impulses to be deconvolved.

The extremum of Equation (4) is acquired by derivation of the filter:

(5) $$\frac{d}{df}\left(\frac{t^{T}y}{\left\|y\right\|}\right)=\frac{d}{df}\left(\frac{t_{1}y_{1}}{\left\|y\right\|}\right)+\frac{d}{df}\left(\frac{t_{2}y_{2}}{\left\|y\right\|}\right)+\cdots +\frac{d}{df}\left(\frac{t_{N-L}y_{N-L}}{\left\|y\right\|}\right)=0$$

Equation (5) is transformed as follows:

(6) $$\frac{d}{df}\left(\frac{t^{T}y}{\left\|y\right\|}\right)={\left\|y\right\|}^{-1}\left(t_{1}M_{1}+t_{2}M_{2}+\cdots +t_{k}M_{k}\right)-{\left\|y\right\|}^{-3}t^{T}yX_{0}y=0$$

where ***M****_k_* =[*x*_*k* + *L*−1_, *x*_*k* + *L*−2_, …,*x*_*k*_]*^T^*, then Equation (6) can be converted into the following formulas:

(7) $$\frac{d}{df}\left(\frac{t^{T}y}{\left\|y\right\|}\right)={\left\|y\right\|}^{-1}X_{0}t-{\left\|y\right\|}^{-3}t^{T}yX_{0}y=0$$

(8) $$\frac{t^{T}y}{{\left\|y\right\|}^{2}}X_{0}y=X_{0}t$$

where $y=X_{0}^{T}f$. Assuming ${\left(X_{0}X_{0}^{T}\right)}^{-1}$ exists, the optimal filter and the solutions can be expressed as:

(9) $$f={\left(X_{0}X_{0}^{T}\right)}^{-1}X_{0}t$$

(10) $$X_{0}=\left[\begin{array}{ccccc} x_{L} & x_{L+1} & x_{L+2} & \cdots & x_{N}\\ x_{L-1} & x_{L} & x_{L+1} & \cdots & x_{N-1}\\ x_{L-2} & x_{L-1} & x_{L} & \cdots & x_{N-2}\\ \vdots & \vdots & \vdots & \ddots & \vdots \\ x_{1} & x_{2} & x_{3} & \cdots & x_{N-L+1} \end{array}\right]$$

(11) $$y=X_{0}^{T}f$$

To process the vibration signal by MOMEDA, the target vector ***t*** can be considered as:

(12) $$t_{n}=\omega *\left(\delta_{round\left(T\right)}+\delta_{round\left(2T\right)}+\cdots +\delta_{round\left(nT\right)}\right)$$

where *δ* represents an impulse at sample *n*, *ω* denotes a window function utilized to extend the target vector, and *T* specifies the fault period. To find the appropriate fault period, the multipoint kurtosis (MKurt) is introduced in MOMEDA, which can be expressed as follows:

(13) $$MKurt=\frac{{\left(\sum {}_{n=1}^{N-L}t_{n}^{2}\right)}^{2}\sum {}_{n=1}^{N-L}{\left(t_{n}y_{n}\right)}^{4}}{\sum {}_{n=1}^{N-L}t_{n}^{8}{\left(\sum {}_{n=1}^{N-L}y_{n}^{2}\right)}^{2}}$$

However, the kurtosis is sensitive to the spurious noise spikes, which could lead to misleading indications. To accurately determine the fault period, this work proposes the multipoint envelope L-kurtosis (*MELkurt*). The definition of MELkurt is given by:

(14) $$MELkurt(i)=LK(a_{i}),i=1,2,\ldots ,n$$

where ***a****_i_* represents the Hilbert envelope signal of the output signal ***y****_i_* of MOMEDA by using the target vector ***t****_i_*, *LK*(·) denotes the calculation operator of the L-kurtosis, which has details that can refer to [32], and *n* symbolizes the number of the target vectors.

It is found that the MELkurt spectra may have a trend term in our test. Thus, a baseline correction method proposed in [33] is applied to refine the MELkurt spectra.

**Figure 1:**
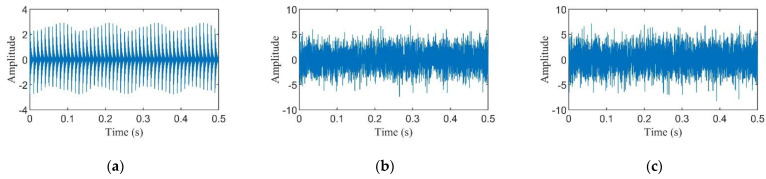
Simulation signal: (**a**) the pure bearing fault impact signal; (**b**) the noise signal; (**c**) the bearing fault composite signal.

**Figure 2:**
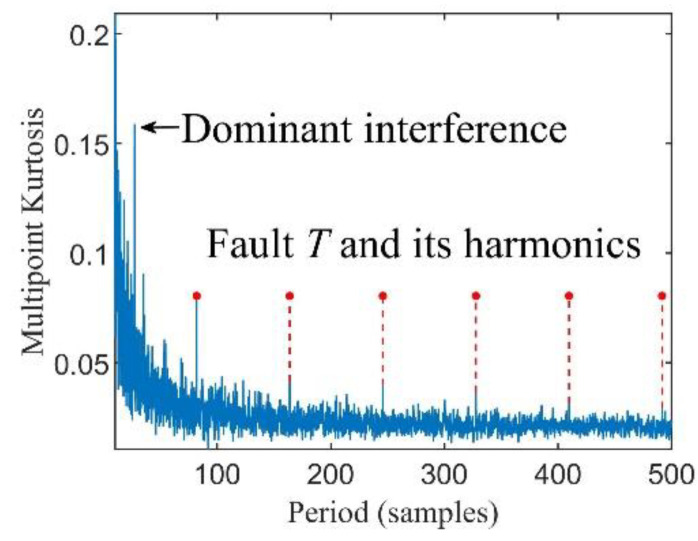
Multipoint Kurtosis spectra.

**Figure 3:**
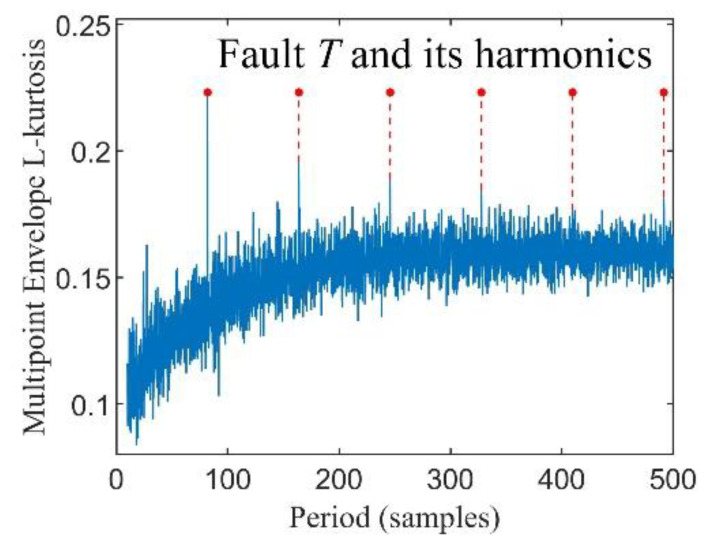
Multipoint Envelope L-kurtosis spectra.

**Figure 4:**
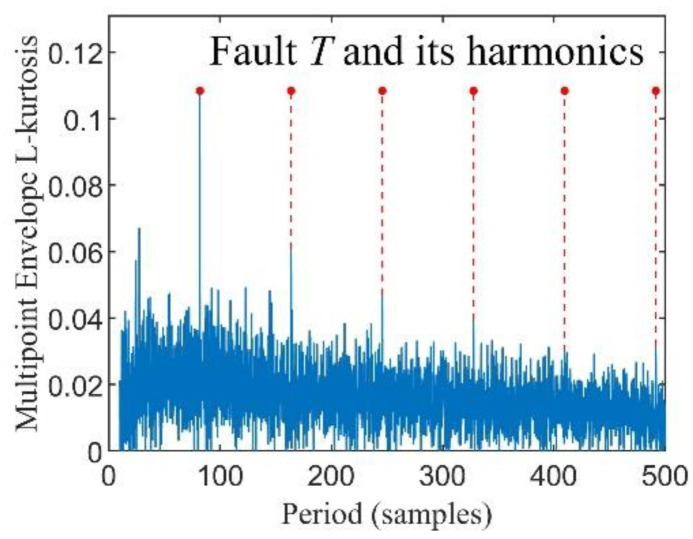
Baseline Correction of MELkurt spectra.

A simulation signal is given as an example, which is expressed as follows.

(15) $$\{\begin{array}{c} x_{1}(t)=3\exp(-350t_{1})\sin(2\pi f_{n}t),t_{1}=\mathrm{mod}(t,1/f_{i})\\ x_{2}(t)=n(t)\\ x(t)=x_{1}(t)+x_{2}(t) \end{array}$$

where *x*_1_(*t*) represents the pure periodic impact signal, as illustrated in Figure 1a, the fault feature frequency *f_i_* of *x*_1_(*t*) is 100 Hz while the excited resonance frequency *f_n_* is 3000 Hz, *x*_2_(*t*) denotes the gaussian white noise whose SNR is −8 dB generated by the ‘awgn’ function of MATLAB, as presented in Figure 1b, and *x*(*t*) is the bearing fault composite signal, as depicted in Figure 1c. Figure 2 shows the result of the simulation signals processed by MKurt, while the result calculated by MELkurt is displayed in Figure 3. As shown in Figure 2 and Figure 3, the peak value of MKurt is not located at the fault period, while the MELkurt can get the peak value at the fault period. It is noted that the multipoint envelope L-kurtosis spectra have a trend term; thus, a baseline correction method is employed to remove it, and Figure 4 demonstrates the result.

### 2.2. Gramian Angular Difference Field

Gramian Angular Difference Field (GADF) is an encoding approach to convert 1D vibration signals into images [10]. Given a vibration signal ***X*** = {*x*_1_, *x*_2_,…, *x_k_*} including *k* values, the signal ***X*** is scaled to [−1,1] interval, firstly, by the function below:

(16) $${\tilde{x}}_{i}=\frac{\left(x_{i}-\max\left(X\right)\right)+\left(x_{i}-\min\left(X\right)\right)}{\max\left(X\right)-\min\left(X\right)}$$

Secondly, the rescaled value ${\tilde{x}}_{i}$ is transformed into polar coordinates. Specifically, the angle *ϕ* is obtained by computing the value of the time series, and the radius *r* is acquired by computing the time stamp, as expressed in Equation (17).

(17) $$\{\begin{array}{l} \phi =\arccos({\tilde{x}}_{i}),-1\leq {\tilde{x}}_{i}\leq 1\\ r=\frac{t_{i}}{M} \end{array}$$

where *t_i_* is the time stamp, and *M* represents a constant factor. The method that maps the time series to the polar coordinate system with only one result is bijective, and the polar coordinates maintain the absolute temporal relations.

Finally, the calculation matrix of GADF that enable the identification of the temporal correlation between different time intervals can be gained by computing the sine value of the trigonometric difference between each point. The GADF matrix is shown as follows:

(18) $$GADF=\left[\begin{array}{ccc} \sin\left(\phi_{1}-\phi_{1}\right) & \cdots & \sin\left(\phi_{1}-\phi_{k}\right)\\ \sin\left(\phi_{2}-\phi_{1}\right) & \cdots & \sin\left(\phi_{2}-\phi_{k}\right)\\ \vdots & \vdots & \vdots \\ \sin\left(\phi_{k}-\phi_{1}\right) & \cdots & \sin\left(\phi_{k}-\phi_{k}\right) \end{array}\right]$$

## 3. Conditional Super Token Transformer

In previous studies, transformer-based deep learning methods have achieved outstanding performance in the natural language processing field. Recently, Vision Transformer (ViT) has extended the transformer-based methods to vision tasks. The ViT model utilizes the self-attention mechanism to capture and incorporate the feature information of the image and outperforms the traditional convolutional neural network with fewer parameters.

### 3.1. Vision Transformer Framework

**Figure 5:**
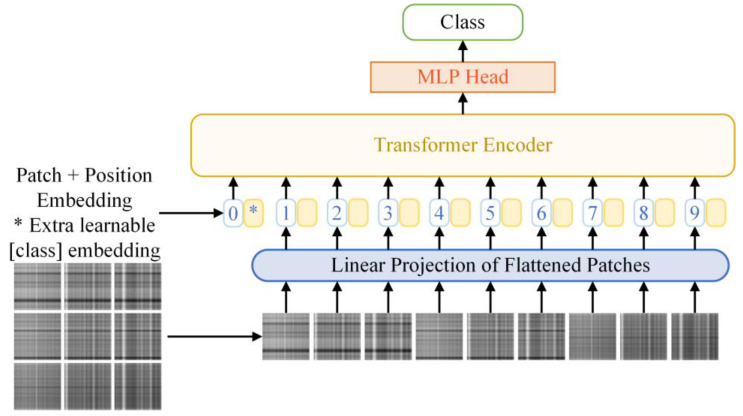
Structure of Vision Transformer.

The basic structure of the Vision Transformer model is expressed in Figure 5. The input image *x* ∈ R^*H* × *W* × *C*^ is split into *N* non-overlapping patches $x_{p}\in R^{N\times (P^{2}\times C)}$. The sequence of patches is flattened into vectors and mapped to *D* dimensions by a trainable embedding matrix *E*. After that, a learnable embedding *x_cls_* is employed to the embedded patches before going through the transformer encoder. In addition, the position embedding *E_pos_* is added to keep position information. The process is expressed by the formula given below:

(19) $$z_{0}=\left[x_{cls};x_{p}^{1}E;x_{p}^{2}E;\cdots ;x_{p}^{N}E\right]+E_{pos},\;E\in R^{(P^{2}\times C)\times D},\;E_{pos}\in R^{(N+1)\times D}$$

where *z*_0_ represents the input of the following transformer encoder.

**Figure 6:**
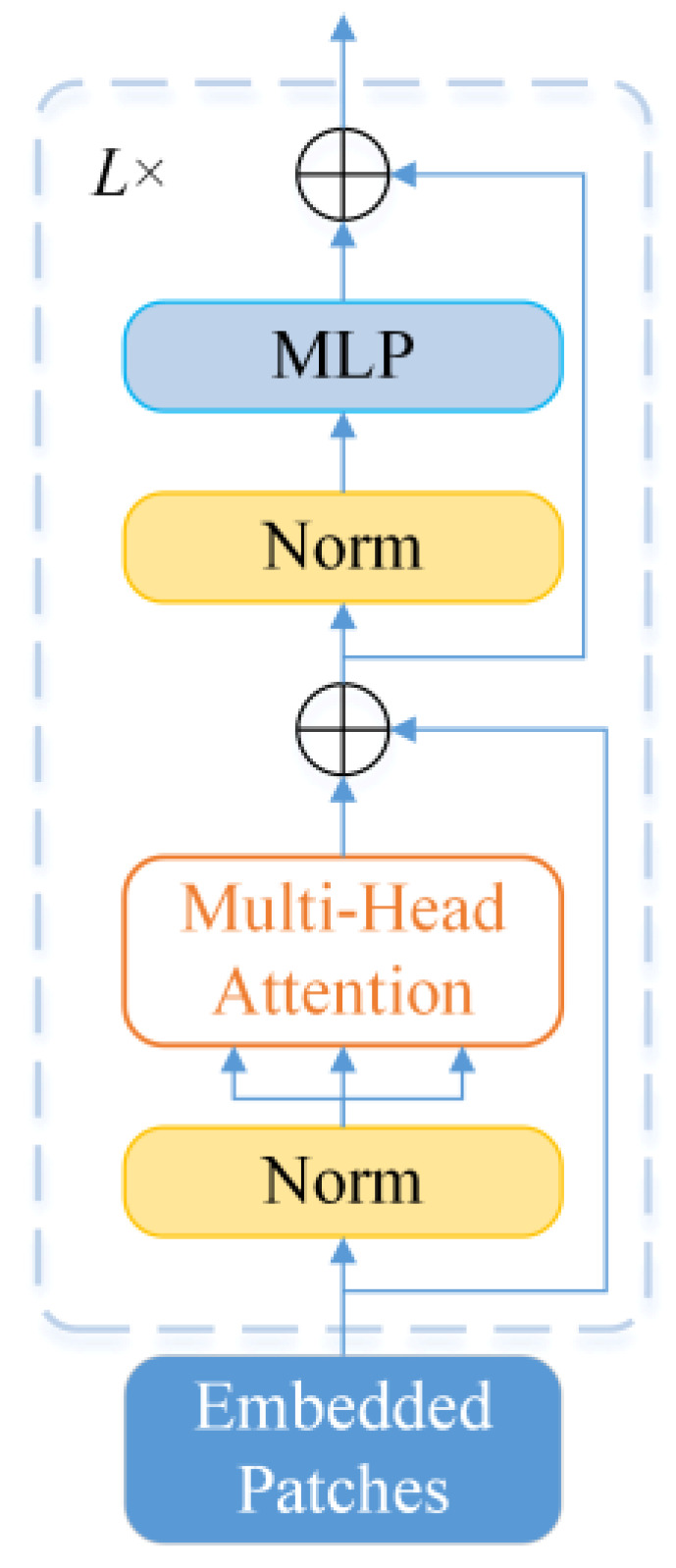
Structure of the Transformer Encoder.

As illustrated in Figure 6, the tokens are then fed into a transformer encoder, which is composed of *L* alternating layers. Specifically, each layer is comprised of a multiheaded self-attention (MSA) block, a multilayer perceptron (MLP) block, and a Layernorm (LN). The output of the encoder is used as the image category representation *y*. The calculation of the transformer encoder is expressed by the formulas below:

(20) $${z^{\prime }}_{\ell }=MSA\left(LN\left(z_{\ell -1}\right)\right)+z_{\ell -1},\;\ell =1\ldots L$$

(21) $$z_{\ell }=MLP\left(LN\left(z_{\ell }^{\prime }\right)\right)+z_{\ell }^{\prime },\;\ell =1\ldots L$$

(22) $$y=LN\left(z_{\ell }\right)$$

The MSA block in the encoder is the core of the transformer. Firstly, the input sequence is linearly projected to obtain queries ***Q***, keys ***K***, and values ***V***. After that, the attention weight *A* is acquired by Equation (23), and the result of the self-attention layer is calculated using Equation (24).

(23) $$A\left(K,Q\right)=\mathrm{softmax}\left(QK^{T}/\sqrt{d}\right)$$

(24) Attention(Q,K,V)=A(K,Q)V

where $\sqrt{d}$ represents the scaling factor.

### 3.2. Improvement Mechanisms

Despite the Vision Transformer method having achieved a remarkable performance for vision tasks, it is weak in establishing links between different local areas. Firstly, we introduce an isotropic architecture called the Super Token Transformer block and adopt window-based self-attention. Meanwhile, a trainable super token is utilized to learn local information in the corresponding window. Secondly, a Super Token Mixer (STM) module is introduced to implement the global information interaction in this paper. Thirdly, a Conditional Positional Encoding (CPE) mechanism is incorporated into the designed model to enhance the generalization ability. CPE enables the model to process input images of different resolutions.

**Figure 7:**
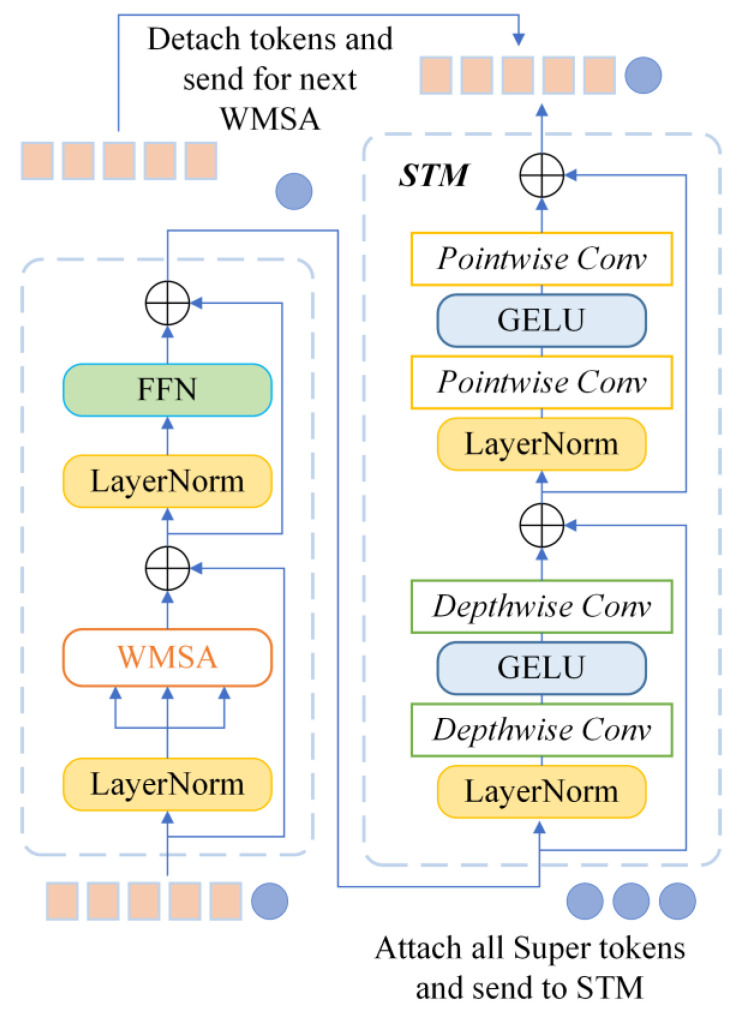
Structure of STT block.

The overall structure of the Super Token Transformer (STT) block is depicted in Figure 7. As shown in Figure 7, two successive blocks constitute the Super Token Transformer. Firstly, the input tokens are fed into the LayerNorm (LN) module and computed by the window-based multihead self-attention (WMSA) module. Secondly, each token is processed with the LayerNorm (LN) module and the feed-forward network (FFN) module. In addition, a residual connection is utilized around each of the two modules. The following Super Token Mixer (STM) block is employed for global information interactions. Therefore, the STT block constructs a local–global feature interactions mechanism. The calculation of the STT block can be described as follows:

(25) $$z_{l}=[SupTk_{l}∥DataTk_{l}]$$

(26) $$z^{\prime }=z_{l}+diag\left(\lambda_{l,1},\cdots ,\lambda_{l,d}\right)\times \mathrm{WMSA}\left(\mathrm{LN}\left(z_{l}\right)\right)$$

(27) $$z^{\prime }=z^{\prime }+diag\left({\lambda^{\prime }}_{l,1},\cdots ,{\lambda^{\prime }}_{l,d}\right)\times \mathrm{FFN}\left(\mathrm{LN}\left(z^{\prime }\right)\right)$$

(28) $$SupTks^{\prime }=\mathrm{STM}\left(SupTks^{\prime }\right)$$

(29) $$z_{l+1}=[SupTk^{\prime }∥DataTk^{\prime }]$$

where *λ_l,I_* and *λ′_l,i_* are learnable weights whose purpose is to scale each information channel dynamically.

In order to perform global information interaction and reduce computational complexity, Super Token Mixer (STM) applies separable convolution to interact across different windows with the locally learnt Super tokens. In this block, the input tokens are computed by two depth-wise convolutions firstly. The information is interchanged across tokens by calculating each channel individually. Then, two point-wise convolutions are employed to enable information interaction on all feature channels at each spatial location. Moreover, two residual connections are utilized between two convolutional blocks. The STM block can be expressed as:

(30) $$x_{in}=[SupTk_{1}∥SupTk_{2}∥\cdots SupTk_{N_{s}}]$$

(31) $$x^{\prime }=x_{in}+C_{DW2}\left(GELU\left(C_{DW1}\left(LN\left(x_{in}\right)\right)\right)\right)$$

(32) $$x_{out}=x^{\prime }+C_{PW2}\left(GELU\left(C_{PW1}\left(LN\left(x^{\prime }\right)\right)\right)\right)$$

where C*_DW_*_1_ and C*_DW_*_2_ represent the two depth-wise convolutions. C*_PW_*_1_ and C*_PW_*_2_ indicate the two point-wise convolutions.

Due to the self-attention being permutation-invariant, which neglects the positional information in tokens, positional encoding methods are applied widely to retain positional information. In previous studies, the flexibility of a transformer cannot be effectively extended by adding the absolute positional encoding (APE) to each token. Meanwhile, the APE is unable to handle the input sequences of different lengths and ignores the translation-invariance. Therefore, the APE significantly restricts the generalization ability of the model. Additionally, the relative positional encoding (RPE) is employed in some research. Although the RPE solves the problem of translation-invariance, it brings extra computational costs and changes the operations in the transformer. Compared with the above methods, the conditional positional encodings (CPE) are produced dynamically and maintain translation-invariance. The CPE can adapt to various input sizes based on the local neighborhood of input tokens, which allows for the processing of images with different resolutions.

### 3.3. Conditional Super Token Transformer

**Figure 8:**
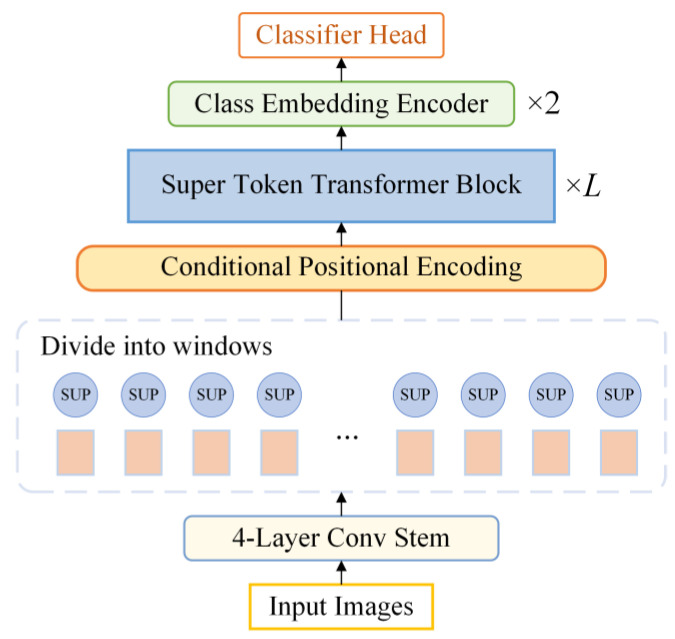
Structure of Conditional Super Token Transformer.

Aiming at automatically learning and extracting features from GADF images, as well as recognizing different bearing working conditions, Conditional Super Token Transformer (CSTT) is developed in this paper. The overall architecture of the Conditional Super Token Transformer (CSTT) is illustrated in Figure 8. First, the input image is initially performed by a four-layer convolutional stem. Then, the output tokens are split into a series of windows with a size of *M* × *M*. After that, each local window gains a learnable token, called Super token (SupTk), to generate (*M* × *M*) + 1 tokens. Meanwhile, the conditional positional encoding is employed to retain the positional information. The processed tokens are fed into the super token transformer block. Finally, a two-layer class embedding encoder is implemented to learn the representation of the image categories. The detailed parameters of CSTT are presented in Table 1.

## 4. The Proposed Method

**Figure 9:**
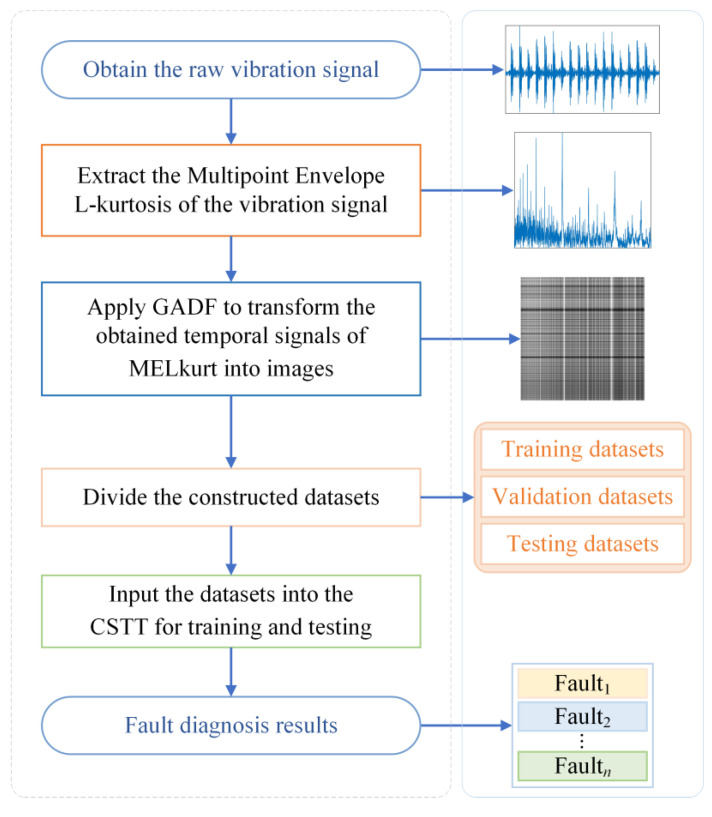
Flowchart of the proposed method.

Based on the MELkurt, GADF, and CSTT methods mentioned above, a novel bearing fault diagnosis framework is established, as demonstrated, in Figure 9. The specific implementation steps of the designed method can be described as follows:

Step 1: Obtain the raw vibration signal datasets with different status.
Step 2: Extract the Multipoint Envelope L-kurtosis of the vibration signal to enhance the fault features.
Step 3: Apply GADF to transform the obtained temporal signals of MELkurt into images and then construct datasets.
Step 4: Divide the datasets into training datasets, validation datasets, and testing datasets.
Step 5: Implement the designed CSTT using the training datasets to identify bearing fault states and obtain the trained model.
Step 6: Evaluate the diagnostic effectiveness of the proposed method on the testing datasets by employing the trained model.

## 5. Experimental Analysis and Results

The evaluation of the proposed CSTT model, in this section, is implemented on the desktop with 3.2 GHz AMD Ryzen 5800H CPU, 16 GB RAM, and NVIDIA GeForce GTX 3070 8 GB GPU under the WIN11 operating system.

### 5.1. Case 1

**Figure 10:**
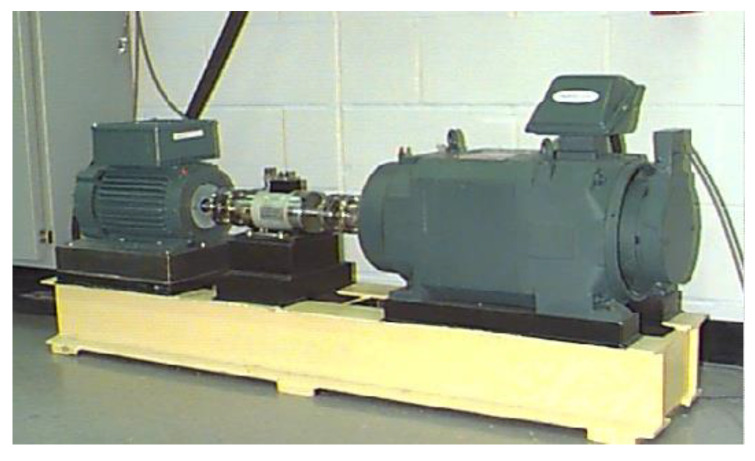
Test-bed of CWRU.

The experimental dataset is acquired from the bearing data center of Case Western Reserve University (CWRU) [34]. The test-bed is shown in Figure 10, which consists of an induction motor, a torque transducer and a dynamometer. In addition, the testing bearing is a deep groove ball bearing SKF6205. Single-point faults are artificially seeded on the outer raceway, inner raceway and ball of the bearings respectively by electro-discharge machining (EDM) technology. Moreover, each fault has three diameters including 0.1778 mm, 0.3556 mm and 0.5334 mm. The selected data is collected at 12 kHz with 1797 rpm motor speed for drive end bearing experiments. According to the configuration of this open dataset, ten kinds of bearing states are selected based on different fault locations and diameters. The details of the dataset composition are shown in Table 2.

**Figure 11:**
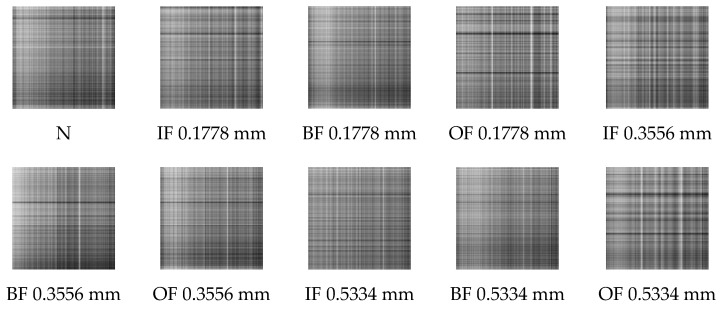
GADF images obtained through MELkurt in Case 1.

**Figure 12:**
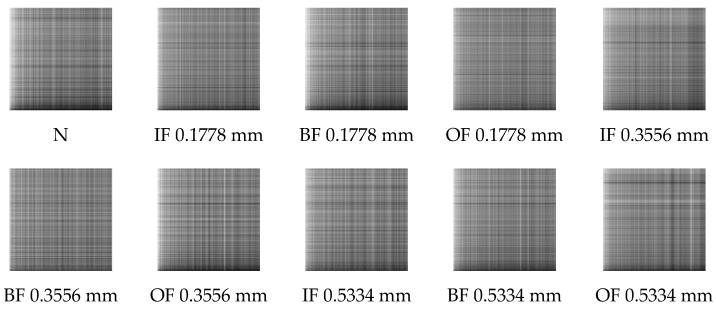
GADF images obtained through MKurt in Case 1.

Firstly, the raw vibration signals are split into sub-sequence signals of equal length, which contain 2048 sampling points. Secondly, the sub-sequence signals are denoised by the method of MOMEDA with MELkurt. Thirdly, the enhanced signals are converted into grayscale images by GADF. Afterwards, GADF image datasets of 10 working conditions are obtained and the results are shown in Figure 11. The GADF image datasets obtained by MOMEDA with MKurt, as comparison, are presented in Figure 12. The cross-validation method is performed to evaluate the recognition ability of the proposed model. Specifically, the obtained images are randomly split into specific quantities, which contain 2000 training samples, 400 validation samples, and 100 testing samples of each fault category. The same random splitting process will be repeated ten times for cross-validation. 

**Figure 13:**
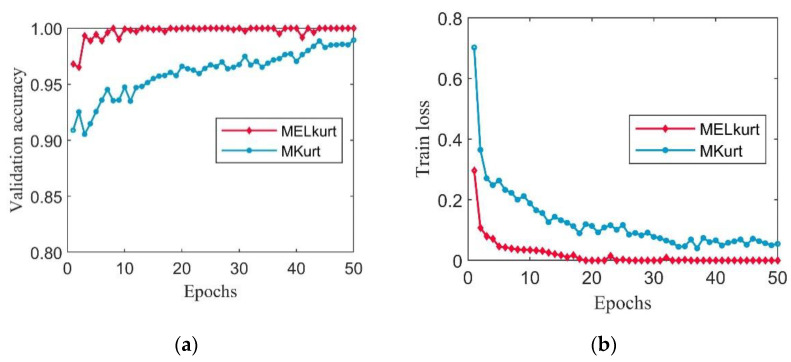
The training process with using MELkurt and MKurt in Case 1: (**a**) validation accuracy curves; (**b**) training loss curves.

To validate the effectiveness and superiority of the MELkurt, the prepared datasets were fed into CSTT. Meanwhile, a pre-trained weight model was introduced into CSTT for transfer learning. The number of iterations was set to 50, and the initial learning rate was 0.001 during the training process. The validation accuracy and training loss are illustrated in Figure 13.

It can be seen from Figure 13 that the datasets processed by MELkurt can achieve stable and accurate recognition after 10 epochs. Moreover, the loss curve of the datasets processed by MOMEDA with the proposed MELkurt method is significantly lower than the datasets processed by MOMEDA with MKurt. To further verify the performance of the designed MELkurt, the trained models of two methods were applied to the corresponding testing datasets. The accuracies and classification standard deviation (Std) of the two methods are demonstrated in Table 3. The standard deviation can be calculated by the formula below:

(33) $$\mathrm{Std}=\sqrt{\frac{1}{N}\sum_{i=1}^{N}{\left(x_{i}-\mu \right)}^{2}}$$

where *x_i_* represents the accuracy of the *i*-th testing sample, *μ* denotes the mean accuracy of all testing samples, and *N* indicates the total number of testing samples.

From Table 3, the maximum classification accuracy of the datasets processed by MELkurt is 100%, and the mean recognition accuracy is 100%. Meanwhile, the results of the proposed method are more stable since the standard deviation is 0. According to the above analysis, it can be seen that the diagnosis results of the datasets processed by MELkurt are better than another method in recognition accuracy and stability. The experimental results indicate that the proposed method MELkurt is more suitable for applying to the bearing fault diagnosis.

**Figure 14:**
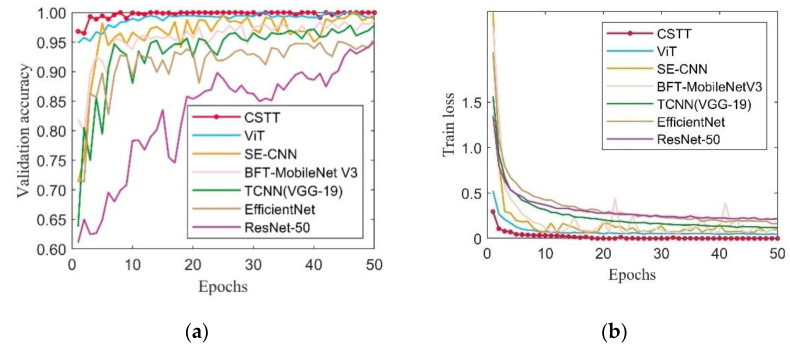
The training process among different models in Case 1: (**a**) validation accuracy curves; (**b**) training loss curves.

To further verify the effectiveness of the proposed model, CSTT was evaluated by comparing with ViT and several CNN-based models, which are SE-CNN, BFT-MobileNet V3, TCNN (VGG-19), EfficientNet, and ResNet-50. Figure 14 demonstrates the validation accuracy and training loss of different models. It can be seen that the validation accuracy of CSTT becomes stable around 100% after reaching 10 epochs. As seen from Figure 14b, the training loss curve of CSTT decreases rapidly around 5 epochs, and then, it slowly drops to a value near zero.

Compared with different CNN-based models, the designed CSTT and ViT show an outstanding convergence speed and tend to be stable in a small number of epochs. Meanwhile, the recognition accuracy of CSTT is highest and the loss is minimum within 50 epochs. The results indicate that the models based on transformer structure are more powerful than CNN-based models in this experiment. 

**Figure 15:**
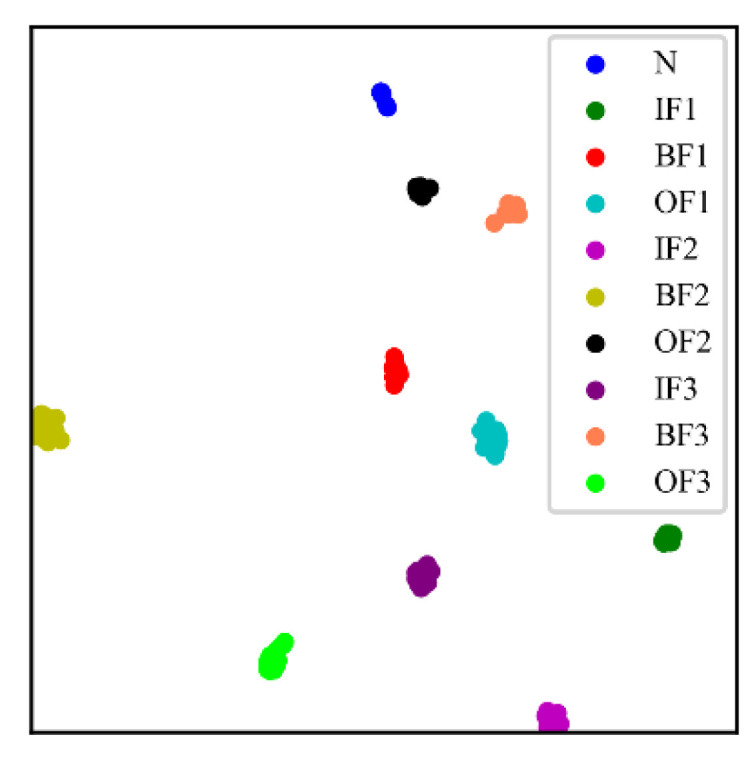
Visualization results of CSTT in Case 1.

To verify the superiority of CSTT among different models, the testing dataset was utilized for further comparisons. The t-distributed stochastic neighbor embedding (t-SNE) method is applied to realize the visualization of the feature learning ability and classification effect of CSTT [35], as shown in Figure 15. As seen in Figure 15, CSTT can effectively extract fault features and identify different fault states.

The comparison of results and the time consumed for diagnosis between CSTT and other models are displayed in Table 4. From Table 4, the CSTT achieves 0.05% average recognition accuracy improvement over ViT, 1.14% over SE-CNN, 1.15% over BFT-MobileNet V3, 1.38% over TCNN (VGG-19), 1.79% over EfficientNet, and 2.48% over ResNet-50. Meanwhile, the standard deviation of CSTT is 0, which indicates the designed method has the best stability compared to other models. In the industrial field, real-time fault monitoring has a high requirement for the efficiency and stability of diagnosis. Seen from Table 4, the designed CSTT takes 3.62 s in the testing process, which outperforms most comparative models with higher testing accuracies. Although the testing time of EfficientNet is less than CSTT, its classification accuracies are lower than CSTT. The experimental results show that the CSTT method has excellent stability and achieves reliable diagnostic accuracy with great efficiency.

### 5.2. Case 2

**Figure 16:**
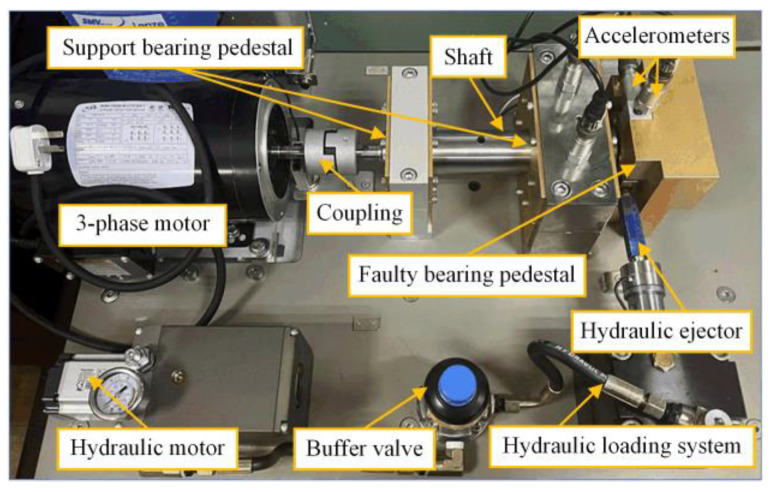
Test-bed of Case 2.

**Figure 17:**
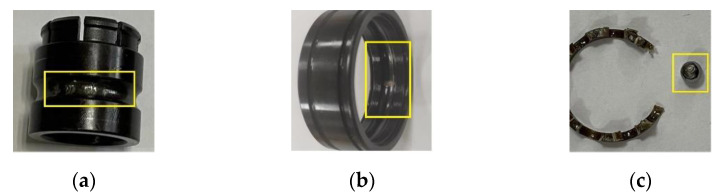
Bearing damage pictures: (**a**) Inner-race fault; (**b**) Outer-race fault; (**c**) Ball fault.

In this case, a new dataset is adopted to further validate the robustness and the generalization ability of the CSTT. The vibration signal acquisition platform is shown in Figure 16, which mainly consists of a three-phase induction motor, hydraulic loading system, normal support bearings, and faulty bearing (stiffened NTN 6205-2RS). The motor works with a speed of 2115 rpm, corresponding to the load of 596 kg. The laser processing technology is utilized to seed different faults in experimental bearings. In addition, the fault types can be divided into ball fault (BF), inner race fault (IF), and outer race fault (OF), as shown in Figure 17. Thus, bearing states mainly contain four categories in this dataset.

**Figure 18:**
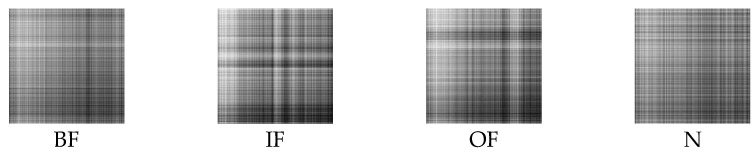
GADF images obtained through MELkurt in Case 2.

**Figure 19:**
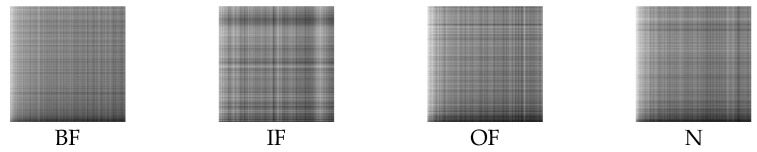
GADF images obtained through MKurt in Case 2.

The analysis process of this case is the same as in Case 1. As in Case 1, the sub-sequence signals of equal length contain 2048 sampling points. Figure 18 displays the GADF images of four bearing states processed by MOMEDA with MELkurt. Figure 19 shows the GADF images obtained through MOMEDA with MKurt. The cross-validation method is also implemented in this case. The obtained images are randomly split, and each bearing working condition has 2000 samples to form a training dataset, 400 samples to form a validation dataset, and 100 samples to form a testing dataset.

**Figure 20:**
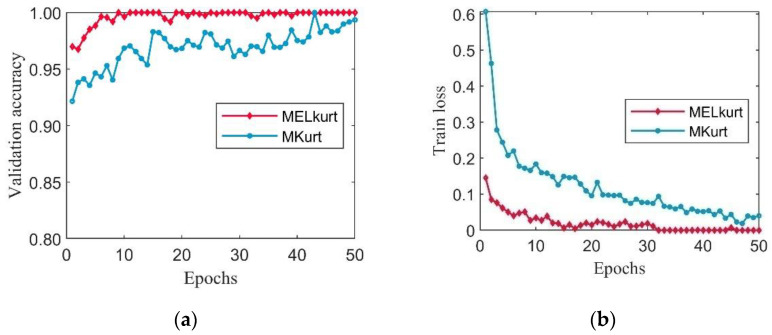
The training process with using MELkurt and MKurt in Case 2: (**a**) validation accuracy curves; (**b**) training loss curves.

In this case study, two prepared datasets are fed into CSTT to verify the effectiveness of the proposed MELkurt. Figure 20 illustrates the validation accuracy and training loss of two methods during the training process.

Similarly, the validation accuracy of the MELkurt method can still quickly become stable by comparing it with the MKurt. At the same time, the loss curve of MELkurt is always lower than that of MKurt in 50 epochs. As is the same for Case 1, the trained models of two methods are employed to process the corresponding testing datasets, and the results are shown in Table 5.

From Table 5, the mean accuracy and standard deviation of the MELkurt method are 100% and 0, respectively. These results clearly demonstrate that the designed MELkurt can effectively improve the recognition accuracy and stability of the CSTT model.

**Figure 21:**
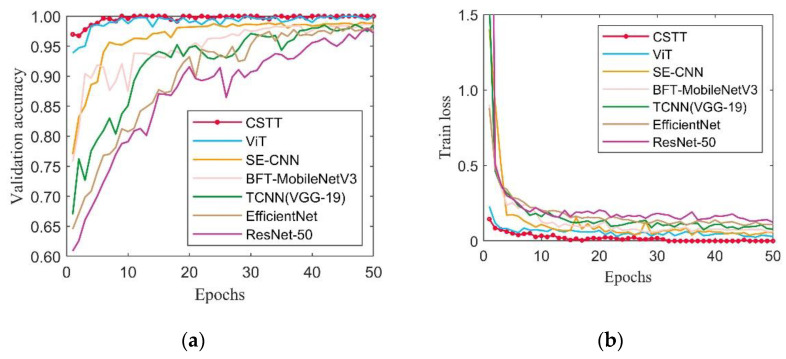
The training process among different models in Case 2: (**a**) validation accuracy curves; (**b**) training loss curves.

**Figure 22:**
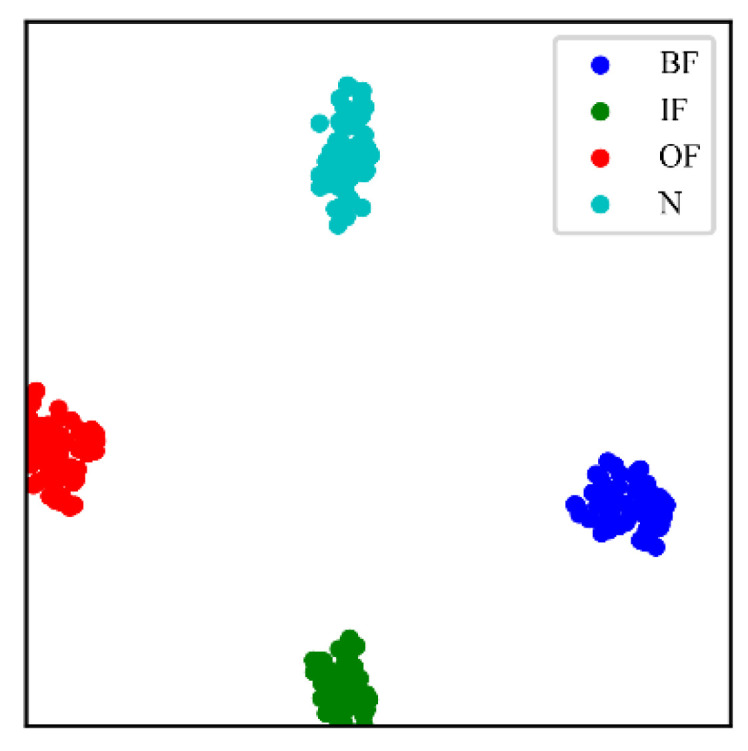
Visualization results of CSTT in Case 2.

The CSTT is still compared with ViT, SE-CNN, BFT-MobileNet V3, TCNN (VGG-19), EfficientNet, and ResNet-50. Figure 21 demonstrates the validation accuracy and train loss of all models. Among these models, the classification of the proposed CSTT achieves the best performance in both accuracy and stability. Table 6 shows the diagnosis results and total time consumed for each model of the testing datasets. The visualization of the feature extraction of CSTT is presented in Figure 22.

Table 6 shows that the classification accuracies of CSTT are greater than ViT and outperform those of CNN-based models. Meanwhile, the proposed CSTT still has an excellent performance in recognition efficiency. These results prove the superior generalization and the robustness ability of the proposed CSTT.

## 6. Conclusions

This work presents a novel deep learning fault diagnosis method of rolling bearing based on MELkurt, GADF, and CSTT. Combined MELkurt with GADF, an enhanced image representation method of vibration signals, is developed in this paper. The designed MELkurt is superior to MKurt for fault signal feature enhancement since the MELkurt is more robust to suppress background noise. The GADF is employed to convert the obtained temporal signals of MELkurt into images without setting internal parameters in advance, which avoids the drawbacks of relying heavily on prior knowledge. Besides, the GADF images can preserve the variable features and the temporal dependency. To effectively and automatically extract the features of the GADF images, the original Vision Transformer (ViT) is improved by incorporating the Super Token Transformer block, Super Token Mixer (STM) module, and Conditional Positional Encoding (CPE) mechanism appropriately, thus proposing the Conditional Super Token Transformer (CSTT). During two experimental datasets, the results showed that GADF image datasets of MELkurt can achieve higher diagnostic accuracy and better stability than the datasets of MKurt. It can be validated that the MELkurt spectra are more suitable for feature visualization. Through comparison with the ViT and several CNN-based models, the proposed CSTT greatly outperforms them, with an average recognition accuracy of 100% and a standard deviation of 0. The proposed method has exhibited an outstanding performance in bearing fault diagnosis, with excellent feature extraction and generalization ability.

In future work, the proposed model will be implemented to diagnose more bearing states with different severity levels. Meanwhile, multimodal information fusion will be considered to further improve the diagnosis accuracy of the proposed method.

## Figures and Tables

**Table 1:** Detailed parameters of CSTT.

Layers	Input Size	Window Size	Heads
25	224 × 224	7 × 7	8

**Table 2:** The composition of the dataset.

Bearing State	Data Number	Fault Size (mm)	Label
Normal (N)	97	-	N
Inner-race fault (IF)	105	0.1778	IF1
	169	0.3556	IF2
	209	0.5334	IF3
Ball fault (BF)	118	0.1778	BF1
	185	0.3556	BF2
	222	0.5334	BF3
Outer-race fault (OF)	130	0.1778	OF1
	197	0.3556	OF2
	234	0.5334	OF3

**Table 3:** The testing results using MELkurt and MKurt in Case 1 (%).

Methods	Max	Min	Mean	Std
MELkurt	100.00	100.00	100.00	0
MKurt	99.03	97.48	98.32	0.23

**Table 4:** The results of the testing dataset among different models in Case 1 (%).

Methods	Max	Min	Mean	Std	Testing Time (s)
Conditional Super Token Transformer (CSTT)	100.00	100.00	100.00	0	3.62
Vision Transformer (ViT)	100.00	99.91	99.95	0.03	5.32
SE-CNN	99.25	98.52	98.86	0.18	4.12
BFT-MobileNetV3	99.21	98.46	98.85	0.26	5.71
TCNN (VGG-19)	98.94	98.33	98.62	0.24	6.75
EfficientNet	98.79	97.54	98.21	0.43	3.28
ResNet-50	98.42	96.43	97.52	0.89	4.05

**Table 5:** The testing results using MELkurt and MKurt in Case 2 (%).

Methods	Max	Min	Mean	Std
MELkurt	100.00	100.00	100.00	0
MKurt	98.95	97.34	98.15	0.24

**Table 6:** The results of the testing dataset among different models in Case 2 (%).

Methods	Max	Min	Mean	SD	Testing Time (s)
Conditional Super Token Transformer (CSTT)	100.00	100.00	100.00	0	3.74
Vision Transformer (ViT)	100.00	99.84	99.93	0.03	5.03
SE-CNN	98.97	98.42	98.70	0.21	4.96
BFT-MobileNetV3	98.81	98.31	98.55	0.22	5.19
TCNN (VGG-19)	98.73	97.92	98.41	0.11	6.05
EfficientNet	98.59	97.23	98.35	0.52	3.59
ResNet-50	98.68	96.59	97.81	0.92	5.37

## Data Availability

Not applicable.
